# Diagnosis value of targeted and metagenomic sequencing in respiratory tract infection

**DOI:** 10.3389/fcimb.2024.1498512

**Published:** 2024-12-12

**Authors:** Yukun Kuang, Weiping Tan, Chaohui Hu, Zehan Dai, Lihong Bai, Jiyu Wang, Huai Liao, Haihong Chen, Rongling He, Pengyuan Zhu, Jun Liu, Canmao Xie, Zunfu Ke, Ke-Jing Tang

**Affiliations:** ^1^ Department of Pulmonary and Critical Care Medicine, The First Affiliated Hospital, Sun Yat-sen University, Guangzhou, China; ^2^ Institute of Respiratory Diseases of Sun Yat-sen University, Guangzhou, China; ^3^ Guangzhou Kingcreate Biotechnology Co., Ltd., Guangzhou, China; ^4^ Molecular Diagnosis and Gene Testing Center, The First Affiliated Hospital, Sun Yat-sen University, Guangzhou, China; ^5^ Department of Pathology, The First Affiliated Hospital, Sun Yat-sen University, Guangzhou, China; ^6^ Institute of Precision Medicine, The First Affiliated Hospital, Sun Yat-sen University, Guangzhou, China; ^7^ Department of Pharmacy, The First Affiliated Hospital, Sun Yat-sen University, Guangzhou, China

**Keywords:** broad-spectrum pathogen identification, targeted next-generation sequencing (tNGS), metagenomic next-generation sequencing (mNGS), diagnosis value, respiratory tract infection

## Abstract

**Background:**

Targeted next-generation sequencing (tNGS) has become a trending tool in the field of infection diagnosis, but concerns are also raising about its performance compared with metagenomic next-generation sequencing (mNGS). This study aims to explore the clinical feasibility of a tNGS panel for respiratory tract infection diagnosis and compare it with mNGS in the same cohort of inpatients.

**Methods:**

180 bronchoalveolar lavage fluid samples were collected and sent to two centers for mNGS and tNGS blinded tests, respectively. The concordance between pathogen reports of both methods and the clinical significance among samples with/without known etiology was further evaluated.

**Results:**

Overall, both methods displayed high agreement on pathogen reports, as the average percent agreement reached 95.29%. But tNGS presented a slightly higher detection rate per species than mNGS (P_Wilcoxon_=1.212e-05; standard mean difference = 0.2887091), as detection rates for 32 out of 48 species were higher than those of mNGS. Due to limitations of panel coverage, tNGS identified 28 fewer species than mNGS, among which only 3 were considered clinically relevant. In reference to composite reference standard, accuracy, sensitivity, and specificity combining both tNGS and mNGS reached 95.61%, 96.71%, and 95.68%, respectively, while positive prediction value (PPV) was low at 48.13%, which was caused by low agreement regarding opportunistic pathogens. tNGS and mNGS improved the etiology identification in 30.6% (55/180) and 33.9% (61/180) cases, respectively.

**Conclusion:**

Collectively, tNGS presented a similar overall performance in pathogen identification compared to mNGS, but outperformed in some pathogens. This study also demonstrated that deployment of tNGS significantly improves etiology identification in routine practice and provides hints for clinical decisions. The low agreement between clinical diagnosis and NGS reports towards opportunistic pathogens implies that adjudication is essential for report interpretation. Finally, We proposed tNGS as a diagnosis option in clinical practice due to its cost-efficiency.

## 1 Introduction

Respiratory tract infections (RTIs) pose a severe threat to global health, causing great morbidity and mortality on a global scale (Lozano et al., 2012). Severe RTIs may lead to life-threatening pneumonia and acute respiratory distress syndrome. Accurate pathogen identification is essential in clinical practice, as delayed or incorrect diagnosis may result in adverse outcomes, such as misuse of antibiotics, and may worsen the prognosis for patients (Ewig et al., 2002; Bleeker-Rovers et al., 2007; Weng et al., 2017).

Determining the etiology of RTI is usually a formidable task in clinical practice. As an opening to the environment, the respiratory tract is exposed to an extensive list of pathogenic factors, either infectious or non-infectious (Dickson et al., 2016; Man et al., 2017), which sometimes lead to the development of various symptoms, including expectoration, cough, fever, hemoptysis etc. Such symptomatology drives pulmonologists to employ comprehensive settings to identify the etiology in routine practice, during which multiple tests are usually employed, including, but not limited to, microscopic examination, imaging, immunology, and molecular testing. A common limitation of these methods is their narrow spectrum, thus the choice of these methods is made accordingly based on presumed pathogens. But still, employing a selection of tests may fail to confirm the causal factor, and pulmonologists may have to resort to empirical treatment for critically ill patients before etiology is determined.

Next-generation sequencing (NGS) has been proposed as a promising tool for resolving difficulties of identifying etiology (Zhong et al., 2021). NGS could be introduced in parallel with routine laboratory tests (RLT), providing comprehensive reports with candidate pathogens and serving as additional evidence for clinical diagnosis. The values of metagenomic next-generation sequencing (mNGS) in RTI diagnosis have been demonstrated (Dong et al., 2022; Li et al., 2022; Xie et al., 2021; Miao et al., 2018; Li et al., 2018; Yang et al., 2019; Shi et al., 2020; Duan et al., 2021). In recent years, targeted next-generation sequencing (tNGS) based on highly-multiplex-PCR is emerging in China, and presents as a competitor for mNGS in pathogen detection. The most distinctive feature of the tNGS panel is its focus on target organisms with clinical relevance, which also benefits from increased sensitivity to low-abundant pathogens. Target amplification could also eliminate interference from a high host genetic background (Mamanova et al., 2010; Dennis et al., 2022), and this further increases data usage efficiency and reduces costs. A primary concern regarding tNGS is its diagnostic performance, particularly in comparison to mNGS, a similar approach that has garnered substantial recognition. The second question is to what extent these methods can aid in precise etiology determination, especially considering that tNGS has a target spectrum. Unfortunately, the real-world differences between both methods in clinical practice haven’t yet been explored. The answers to these questions should enhance our understanding of these methods and similar technologies, and may potentially improve diagnosis and therapeutic processes.

In this study, a two-center, retrospective, blinded comparison was conducted to evaluate the pathogen detection performance of tNGS and mNGS in clinical samples. A total of 199 bronchoalveolar lavage fluid (BALF) samples from 190 inpatients with respiratory infections were collected, and eligible samples were sent to two commercial companies for tNGS and mNGS testing, respectively. After the generated pathogen reports underwent case-by-case scrutiny, the method-wise consistency, the accuracy of identifying etiology and the clinical significance were evaluated. Technical comparison, potential pitfalls, and clinical applications of both methods were also discussed.

## 2 Methods and materials

### 2.1 Study design

**Figure 1:**
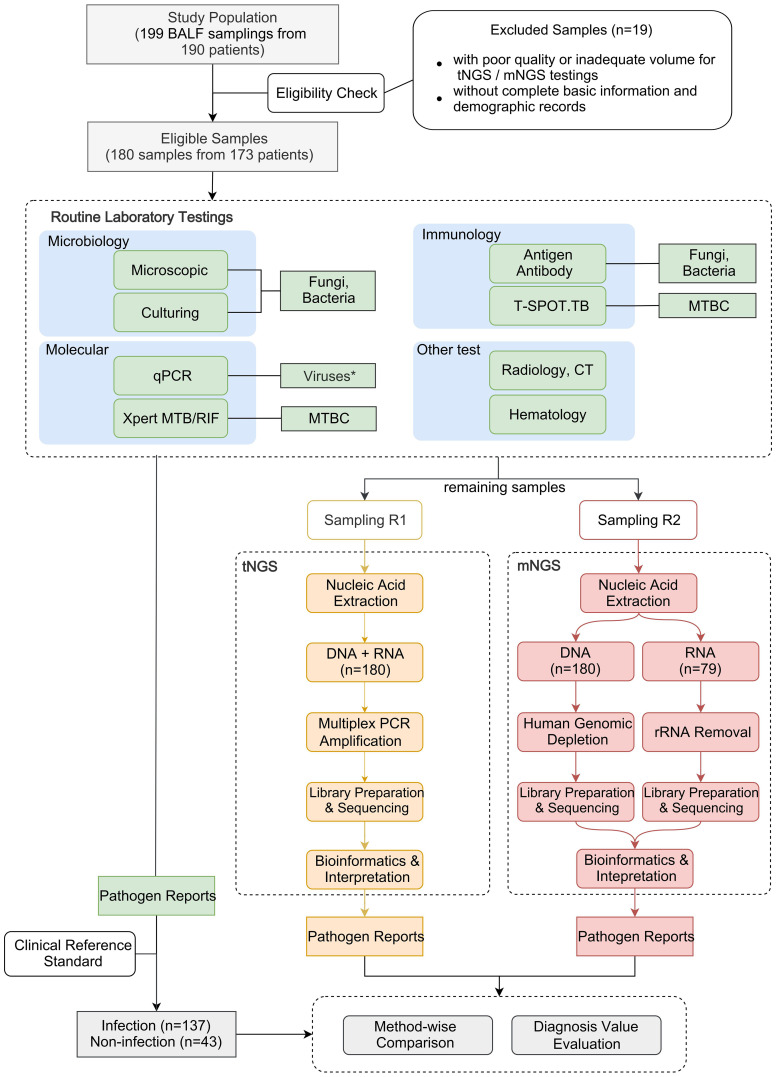
Schematic presentation of the workflow in this study. *qPCR was employed solely for routine virus detection in this study, although it can be used to detect all types of microorganisms.

The workflow of this study is depicted in **Figure 1**. A total of 199 BALF samples were collected from 190 patients admitted to the Department of Pulmonary and Critical Care Medicine, First Affiliated Hospital of Sun Yat-sen University (FAH-SYU). The test results of routine laboratory tests, combined with all information collected during all medical procedures, served as a clinical reference standard for pulmonologists to make a conclusive diagnosis. The remaining samples were stored at -80°C until May 2023, divided into two parts, namely R1 and R2, and sent individually to Kingmed Diagnostics Group Co., Ltd., Guangzhou and VisionMedical Co., Ltd., Guangzhou for tNGS and mNGS. Eligibility criteria: 1) all patients presented typical symptoms of RTI; 2) remaining samples were qualified and sufficient for sequencing; 3) with complete basic information and demographic records. Due to the limitations of mNGS, DNA and RNA samples were extracted and sent for library construction, respectively. Both companies produced pathogen reports independently without knowledge of each other’s and results of any routine laboratory tests until the completion of all data collection. Qualified tNGS and mNGS pathogen reports underwent downstream comparative analysis and evaluation of diagnosis value.

### 2.2 Routine laboratory testing and clinical diagnosis

Different tests were conducted as part of standard routine practice. These tests were selectively conducted based on pathogen suspicion or for exclusion purposes. Results serve as evidence to assist in making a diagnosis. Regular laboratory tests were conducted as part of standard clinical practice. This included conventional microbiological tests (CMTs), such as microbial culture and bacterial and fungal stains, as well as immunological and molecular tests (e.g., qPCR) for pathogen identification and disease diagnosis. These tests were performed by the Department of Laboratory Medicine at FAH-SYU. In addition, acid-fast bacilli staining and culture, Xpert MTB/RIF, and T-cell-based tests for tuberculosis infection (T-SPOT.TB) were also performed for cases with clinical suspicion of *Mycobacterium tuberculosis* (MTB). Each of these methods was developed for the identification of specific types of pathogens and was utilized based on the individual cases. For practical considerations, these tests were selectively conducted based on clinical suspicion and were not used indiscriminately on all samples. Thus, data of specific methods in some samples may not be available, but results of at least two microbial tests were employed for all samples. Intermediate results of individual methods would not be considered positive unless consistency was observed from at least two methods. Results of all tests were combined and taken into consideration to conclude a diagnosis. The positive calling criteria for each method are summarized in **Supplementary Table 1**.

A composite reference standard for etiology identification was established to serve as a criterion for physicians to make initial clinical diagnoses. In brief, two experienced physicians independently reviewed the medical records, including the chart interview process, routine laboratory test results, treatment responses and other information from all medical procedures, to determine whether the patients had infections or non-infections and to identify potential causal pathogens. Disagreements between physicians were resolved through in-depth discussions, during which senior physicians participated until a consensus was reached.

### 2.3 Targeted NGS pathogen detection

Samples were sent to Kingmed Diagnostics Group Co., Ltd., Guangzhou, China for tNGS sequencing. In brief, the development of the tNGS panel first started with a rigorous and exhaustive survey of different types of reference materials, including academic papers, books, and expert consensus, to determine the target spectrum. A corresponding reference database consisting of representative sequences of these microbes described above was curated from NCBI NT database. For tNGS, the panel was designed to cover a spectrum of 153 microorganisms, including 68 viruses, 65 bacteria, 14 fungi, 3 chlamydiae, and 3 mycoplasmas. A full list of all target species was detailed in **Supplementary Table 2**. Primer design was guided by several critical parameters, including GC content, primer length, amplicon size, melting temperature, and potential secondary structures, followed by a specificity check. For more details of it, please refer to Yin’s study (Yin et al., 2024). The BALF samples with high viscosity were diluted 1:1 with 0.1 M dithiothreitol prior to nucleic acid extraction. Automated nucleic acid extraction was performed using MagPure Pathogen DNA/RNA Kit B (Magen Biotechnology, Guangzhou, Guangdong, China) on KingFisher™ Flex Purification System (Thermo Fisher Scientific, Waltham, MA, USA). Nuclease-free water (Invitrogen, Waltham, MA, USA) was used as a non-template control to detect contamination.

Multiplex PCR pre-amplification of target loci and library preparation were performed using a respiratory pathogen test kit, RP100 (KingCreate, Guangzhou, Guangdong, China). Generated libraries were quantified using Equalbit DNA HS Assay Kit (Vazyme Biotech, Nanjing, Jiangsu, China) with an Invitrogen™ Qubit™ 3.0/4.0 (Thermo Fisher Scientific, Waltham, MA, USA) Fluorometer to ensure that all samples had library densities ≥ 0.5 ng/μL, otherwise, they were subjected to library reconstruction. DNA fragment analysis was performed using the Qsep100 Capillary Electrophoresis System™ (BiOptic Inc., Jiangsu, China) and its compatible Standard Cartridge Kit. After library qualification, sequencing was performed using KM MiniSeqDx-CN Sequencing Kit on the sequencing platform KM MiniSeq Dx-CN (KingCreate, Guangzhou, Guangdong, China).

Generated sequencing data was analyzed using a customized bioinformatic workflow. Generated sequencing raw read data underwent quality control procedures. The fastp v0.20.1 (Chen et al., 2018) was employed for adapter trimming and low-quality filtering using default parameters followed by reference-based assembly using Bowtie2 v2.4.1 (Langmead and Salzberg, 2012) in ‘very-sensitive’ mode. For a specific pathogen species or group, the normalized matched read count must reach matched reads per 100,000 reads (RPhK) ≥ 10 to be considered positive. Interpreters conducted the interpretation of the generated pathogen reports, often cooperating with bioinformaticians, before releasing the report.

### 2.4 Metagenomic NGS pathogen detection

Criterion was set up for selecting representative reference sequences of microorganisms from the NCBI NT and NCBI Genome databases. The pathogen list was determined in reference to Johns Hopkins ABX Guide, Manual of Clinical Microbiology, clinical case reports, and peer-reviewed articles, which was detailed in Liu’s study (Liu et al., 2022). Using the QIAamp^®^ UCP Pathogen DNA Kit (Qiagen, Nordrhein-Westfalen, Germany) and following the manufacturer’s instructions, DNA extraction was performed on all samples. Human DNA was eliminated from the samples by using Benzonase (Qiagen, Nordrhein-Westfalen, Germany) and Tween 20 (Sigma, Indiana, U.S.). Total RNA was extracted from the specimens by using the QIAamp^®^ Viral RNA Kit (Qiagen, Nordrhein-Westfalen, Germany) and ribosomal RNA was removed by utilizing the Ribo-Zero rRNA Removal Kit (Illumina, California, USA). The generated cDNA was obtained through reverse transcription (Thermo Fisher Scientific, Waltham, MA, USA).

The Nextera XT DNA Library Prep Kit (Illumina, San Diego, CA, USA) was utilized to create libraries for both the DNA and cDNA samples. The Qubit dsDNA HS Assay Kit (Thermo Fisher Scientific, Waltham, MA, USA) was used to evaluate the quality of the libraries, followed by the High Sensitivity DNA Kit (Agilent, California, USA) on the Agilent 2100 Bioanalyzer (Agilent, California, USA). Each library was subjected to 75 cycles of single-end sequencing on an Illumina NextSeq 550Dx Sequencer (Illumina, San Diego, CA, USA) to generate approximately 20 million reads. Deionized water was included in the extraction process as a negative control to function as a non-template control alongside the specimens.

Trimmomatic (Bolger et al., 2014) was used for low-quality read filtering and adapter trimming. The Burrows-Wheeler Aligner software (Li and Durbin, 2009) was used to exclude reads of human genomes in reference to the human reference genome hg38. For microbial species identification, a curated reference database consisting of > 20000 genomes spanning bacteria, viruses, fungi, protozoa, to other multicellular eukaryotic organisms was employed, as depicted in a previous study (Liu et al., 2022). Microbial reads were aligned to the database using SNAP v1.0 beta.1810 (Zaharia et al., 2011). Positive detection of a given species or genus was reported if the reads per million (RPM) ratio (Miller et al., 2019), or RPM-r, was ≥ 5. The RPM-r was defined as the RPM of the sample divided by the RPM of the negative control. To minimize cross-species misalignments among closely related microorganisms, a penalty mechanism was introduced (Gu et al., 2021). Bioinformaticians conducted the interpretation of the pathogen identification results, generated the pathogen report and approved it before release.

### 2.5 Pathogen report review and refinement

The initial pathogen reports generated from both NGS approaches underwent a pairwise blind review by two experienced physicians. The physicians scrutinized each sample and pathogen report carefully to identify any ambiguous results. After completing this step, the chief researchers unblinded the tNGS and mNGS reports to analyze the results in preparation for the next step. In cases of controversial conclusions concerning any pathogen or patient, a review of the entire workflow was conducted with input from pulmonologists, bioinformaticians, and lab technicians to reach a final conclusion, which was based on the data generated by all methods and sampling.

### 2.6 Clinical value evaluation

To assess the diagnosis value of NGS methods in clinical decision-making and ensure an intuitive understanding, all reports were evaluated by pulmonologists and assigned labels to represent NGS’s contribution to pathogen identification in various scenarios. The five labels used are Agree, Support/Extend, Disagree, Unlikely, and Noncontributory. In general, these labels express agreement on the reports grouped by disease status and qualify whether NGS aided in identifying etiology. Agree and Support/Extend present positive attitudes on cases of infectious samples, while Disagree and Unlikely present disagreement, and Noncontributory presents an intermediate attitudes towards clinically insignificant microbes. The criteria for each label are described in detail in **Table 1**.

**Table 1:** Criteria for determining the diagnosis value of NGS methods.

Value	Type	Etiology	Clinical Presumption	NGS Report	NGS-DTP Match^*^	Criteria Description
Agree	infectious	determined	species level	+	yes	NGS concluded the potential pathogens identified through DTP.
	non-infectious	unknown	−	−	yes	For non-infectious cases determined through DTP, NGS reported no potential pathogens.
Support/Extend	infectious	suspected	genus level or higher	+	yes	Support: diagnosis has a general presumption of potential pathogen (genus level or higher), potential pathogens reported by NGS were consistent with DTP, and thus considered to be clinically revelant.
		unknown	−			Extend: no pathogen presumption was made in clinical diagnosis, potential pathogens reported by NGS were considered to be consistent with DTP.
Noncontributory	non-infectious	unknown	−	+**^†^**	no	Microbes reported by NGS were clinically insignificant.
Unlikely	infectious	determined	species level or higher	+	no	None of the microbes reported by NGS matched the suspected pathogen identified in the clinical diagnosis or DTP.
	non-infectious	unknown	−	+**^‡^**	no	The clinical diagnosis was non-infectious. However, the “potential pathogens” reported by NGS were either considered highly pathogenic based on clinical experience or documented as acute pathogens (e.g. *Mycobacterium tuberculosis*), and these pathogens did not match DTP.
Disagree	infectious	determined	species level	−	no	For infectious cases in which potential pathogens were identified through DTP, NGS failed to detect any microorganisms.

*DTP, Diagnostic and therapeutic process. NGS-DTP Match indicates whether the reported organisms matched DTP.

“+” indicates either clinical diagnosis has determined potential pathogen or at least one pathogen was reported by NGS.

“†” and “‡” indicate that the reported pathogen candidates in cases labeled as “Noncontributory” and “Unlikely” are opportunistic pathogens and acute pathogens, respectively.

“-” indicates no pathogens.

### 2.7 Statistical analysis

Wilcoxon test and Kruskal Wallis test were used to test the pairwise variance for all quantitative measurements (Andrews, 1954). The normal distribution of data was determined by the Shapiro-Wilk test prior to t-test (Shapiro and Wilk, 1965). Linearity between two variables was checked using the *Spearman* rank correlation test (Fieller et al., 1957). These analyses were all conducted using the corresponding functions implemented in base R 4.1.1. The standard mean difference (SMD) was used to measure the degree of variance given two groups of variables with the assistance of the R package *smd* (https://rdrr.io/cran/smd/). A SMD value of “0.20 ≤ estimate ≤ 0.50” is considered to indicate a small effect size (Faraone, 2008).

Positive percent agreement (PPA, percent of the concordant positives), negative percent agreement (NPA, percent of the concordant negatives), sensitivity, specificity, accuracy, positive predictive value (PPV), negative predictive value (NPV), and 95% confidence interval (CI) were all manually calculated following the formulas described in previous studies (Altman and Bland, 1994a, Altman and Bland, 1994b; Bland and Altman, 1996; van Stralen et al., 2009). Percent agreement, PPA and NPA were used for evaluating the agreement of measurement between two NGS approaches. To estimate the clinical agreement of these qualitative tests, the conclusion of clinical diagnosis was used as the reference to determine true positives, true negatives, false positives, false negatives. Based on these, specificity, sensitivity, and accuracy were further calculated.

### 2.8 Sample size estimation

Sample size was estimated as below:


$$N=\frac{{\left(Z_{\alpha }^{C}+Z_{\beta }^{C}\right)}^{2}}{d^{2}}$$

In this formula, *N* represents the total sample size, *C* denotes confidence level, *Z_α_* is the critical value of the standard normal distribution corresponding to the significance level *α*, *β* represents the power level, and *Z_β_* is the critical value of the standard normal distribution corresponding to the power level. The effect size *d*, computed as minimum mean difference divided by standard deviation.

Assuming a confidence level of 0.99, the value of *Z_α_* is 2.576. Assuming a power level of 0.95, the value of *Z_β_* is 1.645. Based on preliminary experimental data (not published), the estimated effect size is 32%. The minimum required sample size is calculated as:


$$N=\frac{{\left(2.576+1.645\right)}^{2}}{0.31^{2}}\approx 173$$

The estimated sample size is 173. Considering the possible insufficiency of remaining samples and possibility of unqualified sequencing test, additional 10% was added to this estimate. The final expected sample size is 190.

## 3 Results

### 3.1 Baseline demographics and clinical characteristics

A total of 199 samples were collected from 190 patients with suspected pneumonia, among which 180 eligible samples passed the enrollment criteria and were included in the following analysis. All samples were randomly selected and collected between July 2021 and October 2022. Although different types of samples were collected for routine clinical practice, only BALF samples were selected for this study. Basic information of the study population is summarized in **Table 2**. All the patients included were over 18 years old, with ages ranging from 19 to 89 (**Supplementary Figure 1**). The majority of study subjects (over 75%) were aged between 40 and 80. 68% of the selected patients were male and 32% were female. All patients had typical pneumonia symptoms. The proportions of patients with cough, expectoration, fever, and hemoptysis account for 72.22%, 52.78%, 39.44%, and 10.00%, respectively (**Table 2**). Some patients had chronic diseases, such as diabetes, cardiovascular disease, hypertension, and issues in other organs. Patients with cardiovascular disease and hypertension represent the highest proportions (29.44% and 31.67%). BALF samples were first taken from these patients for selected laboratory tests to serve as part of routine practice for helping pulmonologists with decision making. After hospitalization/administration of all inpatients, the remaining samples were sent for nucleic acid extraction to facilitate downstream NGS analysis

**Table 2:** Characteristics and history of patients in the study (n=173).

Examinations	Reference Range	Measurements
Clinical manifestation (count; percentage)		
Cough	–	130 (72.22%)
Expectoration	–	95 (52.78%)
Fever	–	71 (39.44%)
Hemoptysis	–	18 (10.00%)
Dyspnea	–	1 (0.56%)
Chest tightness	–	1 (0.56%)
Hematology parameters (median; lower/upper quartile)		
White cell count (× 10^9/L)	3.50-9.50	8.26 (5.46-11.23)
Platelet count (× 10^9/L)	100-300	251 (176-335)
Neutrophil (%)	45.0-75.0	75.5 (64.8-83.8)
Lymphocyte (%)	20.0-50.0	14.4 (7.9-23.6)
Eosinophil (%)	0.5∼5	9 (2-19)
Hemoglobin (g/L)	115-150	118 (99-138)
Plateletcrit (mg/ml)	<0.1	0.09 (0.05-0.26)
C-reactive protein (mg/L)	<5.0	26.93 (7.68-107.09)
Underlying Disease (count; percentage)		
Hypertension	–	57 (31.67%)
Cardiovascular disease	–	53 (29.44%)
Cerebrovascular disease	–	37 (20.56%)
Diabetes	–	28 (15.56%)
Chronic liver disease	–	27 (15.00%)
Chronic kidney disease	–	14 (7.78%)
Chronic obstructive pulmonary disease	–	10 (5.56%)

### 3.2 Method-wise concordance between tNGS and mNGS

**Figure 2:**
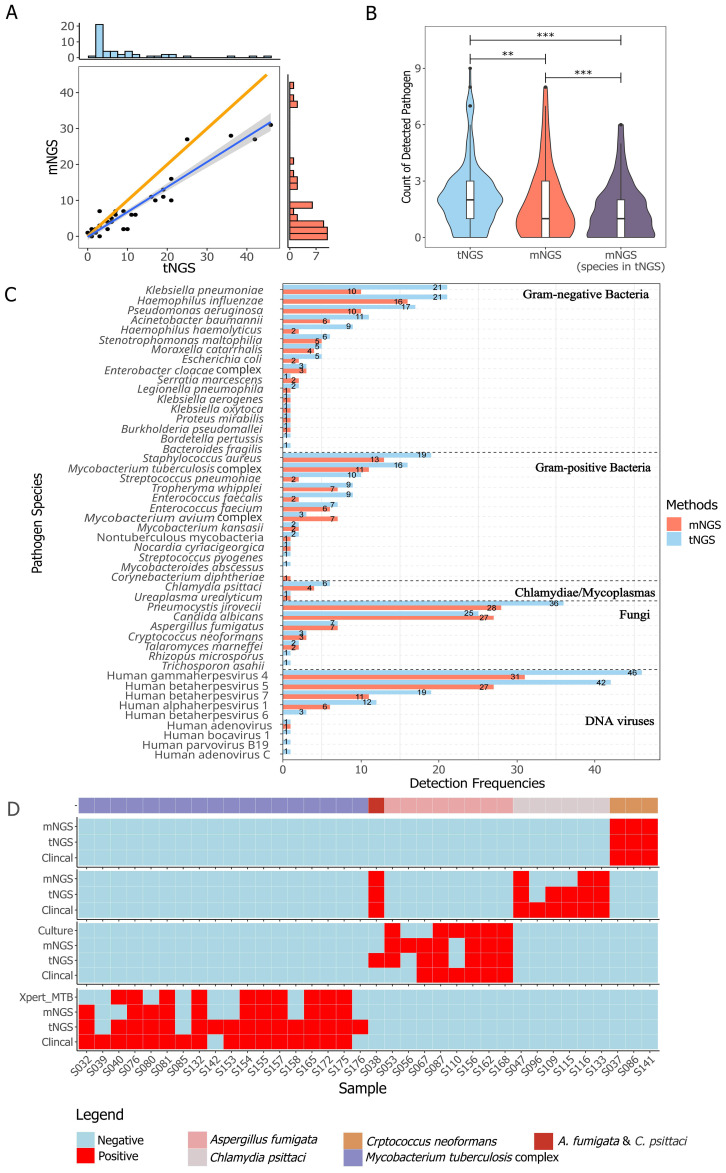
Method-wise comparison of pathogen detection. **(A)** The correlation of identified pathogens between tNGS and mNGS is illustrated with a highlighted blue regression line to emphasize the linearity of positive calls, while an orange line represents the reference line of equality. The correlation was determined using Spearman rank test (P < 0.01). **(B)** Counts of detected pathogen species per sample with group-wise statistical differences confirmed by paired Wilcoxon test (P < 0.01). "**" and "***" indicates a P value of ≤0.05 and ≤0,01, respectively. **(C)** The detection frequency of each pathogen with a method-wise statistical difference confirmed by paired Wilcoxon test (P < 0.01). All the pathogens described or taken into analysis are microbes with DNA genome. Only species covered in the tNGS panel and mNGS were included. **(D)** Comparison of different methods for detection of *Aspergillus fumigata*, *Chlamydia psittaci*, *Crptococcus neoformans*, and *Mycobacterium tuberculosis* complex.

The general performance comparison between tNGS and mNGS is summarized in **Figure 2**. A significant positive correlation in detection frequencies per species was observed between tNGS and mNGS (*Spearman* test, P=1.39e-14, **Figure 2A**). However, tNGS reported more positives than mNGS, as evidenced by the deviated fitted line and a correlation coefficient less than 1 (ρ*_spearman_* =0.85, **Figure 2A**). It is interesting that although tNGS has a much narrower spectrum (153 species, detailed in **Supplementary Table 2**) than mNGS (~20,000), tNGS reported significantly more potential pathogens per sample than mNGS. Such a difference was more pronounced when considering only the targets covered by both the tNGS panel and mNGS (P=1.374e-13, **Figure 2B**). Such variance could be further demonstrated at organism level. An overall difference could be observed between tNGS and mNGS regarding detection frequencies (P-value=1.212e-05, Paired Wilcoxon test). In detail, tNGS exhibited higher detection frequencies for 32 organisms, equal detection frequencies for 12 organisms, and lower detection frequencies for 4 organisms when compared to mNGS. Ten species were identified exclusively by either tNGS or mNGS, each appearing in only one sample (**Figure 2C**). Additionally, mNGS exclusively reported 28 species that are not covered in the tNGS panel (**Supplementary Table 3**). However, 25 of them were considered clinically insignificant by composite reference standard. Although the overall percent agreements of all shared targets showed high concordance between both methods (82.78% ~ 100%) (**Supplementary Table 4**), the PPAs of some species were low (e.g. *Mycobacterium avium* complex at 42.86%). These results highlight the differences in detection prevalence between mNGS and tNGS.

### 3.3 Agreement of tNGS and mNGS with clinical diagnosis

In reference to diagnoses, the overall accuracy, sensitivity, specificity, and negative percent value (NPV) of tNGS/mNGS all exceeded 95%, while the overall PPVs were relatively low at 42.09% (CI_95%_: 27.6%~56.6%) and 53.47% (CI_95%_: 38.7%~68.2%) (**Table 3**). Further inspection revealed that this may be due to the predominance of opportunistic pathogens, as PPVs of opportunistic pathogens by tNGS and mNGS were 15.71% and 21.88%, respectively. An extreme case is Human gammaherpesvirus 4, as only 1 positive was considered clinically relevant out of all 46 positives (for all pathogens, see **Supplementary Table 5**). On the other hand, four selected pathogens - *Cryptococcus neoformans*, *Aspergillus fumigata*, *Mycobacterium tuberculosis* complex, and *Chlamydia psittaci* - considered empirically to be highly pathogenic, had high PPVs (66.7% to 100%) (**Table 3**). Agreement of positive results between clinical diagnosis and other methods regarding these species is summarized in **Figure 2D**. tNGS and mNGS confirmed most of the positive results identified by clinical diagnosis and outperformed other methods. Taking *Mycobacterium tuberculosis* complex as an example, tNGS, mNGS and Xpert reported 14, 11, and 10 positives out of 16 clinically positive samples, respectively. This was also observed in *Aspergillus fumigatus*, *Cryptococcus neoformans*, and *Chlamydia psittaci* (**Figure 2D**).

**Table 3:** Detection performance of NGS methods on specific pathogens in reference to clinical diagnosis.

Pathogen	Method	Accuracy%	Sensitivity%	Specificity%	PPV%	NPV%
		(CI_95%_)	(CI_95%_)	(CI_95%_)	(CI_95%_)	(CI_95%_)
*Cryptococcus neoformans*	tNGS	99.44%	100%	99.44%	66.67%	100%
		(96.9%-99.9%)	(15.8%-100%)	(96.9%-99.9%)	(9.4%-99.16%)	(97.9%-100%)
	mNGS	99.44%	100%	99.44%	66.67%	100%
		(96.9%-99.9%)	(15.8%-100%)	(96.9%-99.9%)	(9.4%-99.2%)	(97.9%-100%)
*Aspergillus fumigata*	tNGS	99.44%	100%	99.43%	85.71%	100%
		(96.9%-100%)	(54.1%-100%)	(96.8%-100%)	(42.1%-99.6%)	(97.9%-100%)
	mNGS	99.44%	100%	99.43%	85.71%	100%
		(96.9%-100%)	(54.1%-100%)	(96.8%-100%)	(42.1%-99.6%)	(97.9%-100%)
*Mycobacterium tuberculosis* complex	tNGS	98.89%	93.75%	99.39%	93.75%	99.39%
		(96.0%-99.9%)	(69.8%-99.8%)	(96.7%-100%)	(69.8%-99.8%)	(96.7%-99.9%)
	mNGS	97.22%	68.75%	100%	100%	97.04%
		(93.6%-99.1%)	(41.3%-89.0%)	(97.8%-100%)	(71.5%-100%)	(93.2%-99.0%)
*Chlamydia psittaci*	tNGS	100%	100%	100%	100%	100%
		(98.0%-100%)	(54.1%-100%)	(98.0%-100%)	(54.1%-100%)	(97.9%-100%)
	mNGS	98.89%	66.67%	100%	100%	98.86%
		(96.0%-99.9%)	(22.3%-95.7%)	(98.0%-100%)	(39.8%-100%)	(96.0%-99.9%)
all pathogens	tNGS	94.56%	98.86%	94.52%	42.09%	99.95%
		(91.5%~97.1%)	(97.0%~100%)	(91.4%~97.7%)	(27.6%~56.6%)	(99.9%~100.0%)
	mNGS	96.54%	94.82%	96.70%	53.47%	99.78%
		(94.6%~98.5%)	(90.3%~99.4%)	(94.7%~98.7%)	(38.7%~68.2%)	(99.5%~100.0%)
all pathogens	total	95.61%	96.72%	95.68%	48.13%	99.86%
		(93.9%~97.4%)	(94.2%~99.3%)	(93.9%~97.5%)	(38.0%~58.3%)	(99.7%~100.0%)

PPV, Positive predictive value; NPV, Negative predictive value.

### 3.4 Accessing the diagnosis value of NGS

**Figure 3:**
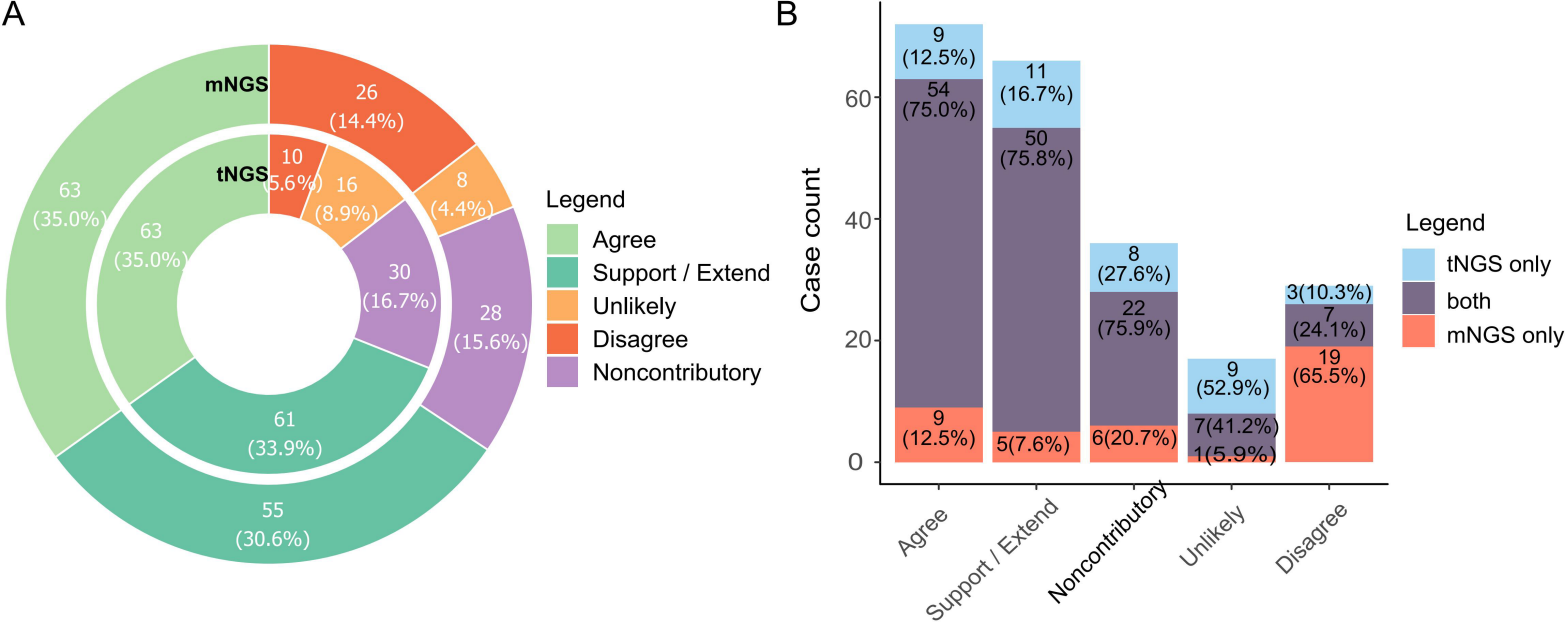
Diagnosis value of NGS methods towards pathogen determination. **(A)** Barplot summarizing cases with different tags of diagnosis value. Pathogen reports were adjudicated by pulmonologists and assigned labels according to the extent they assisted in identifying possible causal pathogens. Label explanation: Agree, NGS reported potential pathogens that was/were consistent with clinical diagnosis (either infectious or non-infectious). Support/Extend, NGS reported unexpected potential pathogens in cases with unknown etiology, and they matched diagnosis (by routine laboratory test) and treatment response. Noncontributory, all microbes reported in non-infectious cases were clinically insignificant. Unlikely, reported microbes did not match diagnosis and treatment response. Disagree, NGS failed to identify any microorganism in infection cases in which important casual pathogens were identified. Full criteria for each label are listed in **Table 1**. **(B)**. Comparison of preference based on value tags between tNGS and mNGS.

To access the diagnosis value of NGS reports, we scrutinized all reports, adjudicated the reported organisms and categorized the reports based on agreement labeling (namely Agree, Support/Extend, Noncontributory, Unlikely, Disagree, see **Table 1** for criteria), assessing whether NGS aided in etiology determination. Tags of Agree and Support/Extend indicate that NGS assisted in identifying potential pathogens, and the total number of reports marked by these two tags reached 124 (68.9%) and 118 (65.6%) for tNGS and mNGS, respectively. In contrast, the total counts of Disagree, Unlikely, and Noncontributory are relatively low, at 31.1% and 34.4%, where most reported pathogens were considered to be clinically insignificant (**Figure 3A**). A preference could be observed between tNGS and mNGS, as the etiology of some cases was identified only by either tNGS or mNGS. tNGS tended to report more cases of Unlikely while mNGS reported more cases of Disagree (**Figure 3B**). We assume that this may be due to sensitivity differences between NGS methods, which will be discussed later.

**Figure 3A** depicts the comprehensive efficacy of NGS in ascertaining etiological factors. Subsequent analyses were conducted on cases where NGS-based determinations of etiology were provided at a report level. We primarily focused on the cases that highlighted the enhanced capability of NGS to identify potential pathogens. At least one NGS method, either tNGS or mNGS, identified the primary pathogen *Mycobacterium tuberculosis*, supported by RLT, in 15 cases. However, no single RLT was capable of detecting *Mycobacterium tuberculosis* in all these instances (**Table 4**). The RLT with the highest number of positives was the Xpert MTB/RIF assay, confirming the pathogen in 10 out of 15 cases, which was less effective than tNGS (14/15), and mNGS(11/15) (**Table 4**). Furthermore, tNGS provided assistance in identifying potential *Mycobacterium tuberculosis* infections in cases where RLT results were negative. Additionally, in four cases, both tNGS and mNGS were instrumental in identifying non-tuberculous mycobacteria to the species level (S075, S085, S120, S149). Regarding the detection of *Aspergillus fumigatus*, it relies on serological tests, culture, and microscopic observation. Serological tests, such as ELISA, depend on an infection window to achieve optimal sensitivity, which may explain the positive results in only three out of the total seven cases. Moreover, the culture method - a time-intensive and comparatively less sensitive approach - yielded positive results in five cases. In contrast, both NGS methods achieved a 100% detection rate (7/7). In six cases, *Chlamydia psittaci* was detected exclusively by NGS methods, with no RLTs providing corroborative evidence. Diagnosis of these cases was definitively attributed to a composite reference standard, reinforced by the clinical context of primary pathogenicity and the patients’ history of frequent poultry exposure.

**Table 4:** Comparison of etiology determined in selected cases by NGS and RLTs.

Sample	Potential Pathogen	Routine Laboratory Test	tNGS	mNGS
RLT-supported etiology				
S032	*Mycobacterium tuberculosis* complex	T-SOPT.TB (+)	tNGS (+)	mNGS (+)
S039	*Mycobacterium tuberculosis* complex	Acid Fast Bacteria Stain (+)	–	–
S040	*Mycobacterium tuberculosis* complex	Xpert MTB (+)	tNGS (+)	–
S076	*Mycobacterium tuberculosis* complex	Xpert MTB (+)	tNGS (+)	mNGS (+)
S080	*Mycobacterium tuberculosis* complex	Acid Fast Bacteria Stain (+)	tNGS (+)	mNGS (+)
S081	*Mycobacterium tuberculosis* complex	Xpert MTB (+)	tNGS (+)	mNGS (+)
S132	*Aspergillus* fungi	*Aspergillus* Elisa (+), fungal fluorescent staining (+), suspected *Aspergillus* hyphae and spores	–	mNGS (+)
	*Penicillium marneffei*	Sputum culture (+), BALF culture (+)	tNGS (+)	mNGS (+)
	*Mycobacterium tuberculosis* complex	Xpert MTB (+)	tNGS (+)	mNGS (+)
S153	*Pseudomonas aeruginosa*	Sputum culture (+), BALF culture (+)	tNGS (+)	mNGS (+)
	*Mycobacterium tuberculosis* complex	Acid Fast Bacteria Stain (+)	tNGS (+)	–
S154	*Mycobacterium tuberculosis* complex	Xpert MTB (+)	tNGS (+)	mNGS (+)
S155	*Mycobacterium tuberculosis* complex	Xpert MTB (+)	tNGS (+)	mNGS (+)
S157	*Mycobacterium tuberculosis* complex	Xpert MTB (+)	tNGS (+)	mNGS (+)
S165	*Mycobacterium tuberculosis* complex	Xpert MTB (+)	tNGS (+)	mNGS (+)
S172	*Mycobacterium tuberculosis* complex	Xpert MTB (+), Acid Fast Bacteria Stain (+)	tNGS (+)	mNGS (+)
S175	*Mycobacterium tuberculosis* complex	Xpert MTB (+), Acid Fast Bacteria Stain (+)	tNGS (+)	mNGS (+)
S176	*Mycobacterium tuberculosis* complex	Acid Fast Bacteria Stain (+)	tNGS (+)	–
S053	*Aspergillus fumigatus*	Sputum culture (+), BALF culture (+)	tNGS (+)	mNGS (+)
	*Staphylococcus aureus*	BALF culture (+)	tNGS (+)	mNGS (+)
S067	*Aspergillus fumigatus*	*Aspergillus* Elisa (+)	tNGS (+)	mNGS (+)
S087	*Aspergillus fumigatus*	*Aspergillus* Elisa (+), fungal fluorescent staining (+)	tNGS (+)	mNGS (+)
S156	*Aspergillus fumigatus*	Sputum culture (+), BALF culture (+), *Aspergillus* Elisa (+)	tNGS (+)	mNGS (+)
S162	*Aspergillus fumigatus*	Sputum culture (+), BALF culture (+)	tNGS (+)	mNGS (+)
S168	*Aspergillus fumigatus*	Sputum culture (+), BALF culture (+)	tNGS (+)	mNGS (+)
S087	*Aspergillus fumigatus*	*Aspergillus* Elisa(+), suspected *Aspergillus* hyphae and spores	tNGS (+)	mNGS (+)
S099	*Streptococcus pneumoniae*	BALF culture (+)	tNGS (+)	–
	*Candida albicans*	BALF culture (+)	tNGS (+)	mNGS (+)
S151	*Candida albicans*	Sputum culture (+), BALF culture (+)	–	mNGS (+)
S145	*Candida albicans*	BALF culture (+)	tNGS (+)	mNGS (+)
S146	*Candida albicans*	BALF culture (+)	tNGS (+)	mNGS (+)
S110	*Enterococcus faecium*	Pleural and peritoneal effusions (+)	tNGS (+)	mNGS (+)
S002	CMV	PCR(+), antibody(+)	tNGS (+)	mNGS (+)
	EB	PCR(+)	–	–
RLT-negative				
S158	*Mycobacterium tuberculosis*	–	tNGS (+)	–
S075	*Mycobacterium avium* complex	–	tNGS (+)	mNGS (+)
S085	*Mycobacterium avium* complex	–	tNGS (+)	mNGS (+)
S120	*Mycobacterium avium* complex	–	tNGS (+)	mNGS (+)
S149	*Mycobacterium kensasii*	–	tNGS (+)	mNGS (+)
S038	*Chlamydia psittaci*	–	tNGS (+)	mNGS (+)
	*Aspergillus fumigatus*	–	tNGS (+)	
S047	*Chlamydia psittaci*	–	tNGS (+)	mNGS (+)
S109	*Chlamydia psittaci*	–	tNGS (+)	–
S115	*Chlamydia psittaci*	–	tNGS (+)	–
S116	*Chlamydia psittaci*	–	tNGS (+)	mNGS (+)
S133	*Chlamydia psittaci*	–	tNGS (+)	mNGS (+)
S098	*Pneumocystis jirovecii*	–	tNGS (+)	mNGS (+)
	Rhinovirus	–	tNGS (+)	–
S074	*Pneumocystis jirovecii*	–	tNGS (+)	mNGS (+)
S064	*Pneumocystis jirovecii*	–	tNGS (+)	mNGS (+)
S012	*Pneumocystis jirovecii*	–	tNGS (+)	mNGS (+)
	HCMV	–	tNGS (+)	mNGS (+)
S019	*Pneumocystis jirovecii*	–	tNGS (+)	mNGS (+)
S026	*Pneumocystis jirovecii*	–	tNGS (+)	mNGS (+)
S104	*Pneumocystis jirovecii*	–	tNGS (+)	mNGS (+)
S107	*Pneumocystis jirovecii*	–	tNGS (+)	mNGS (+)
S128	*Pneumocystis jirovecii*	–	tNGS (+)	–
S177	*Pneumocystis jirovecii*	–	tNGS (+)	mNGS (+)
S101	Rhinovirus	–	tNGS (+)	–
S111	Human metapneumovirus	–	tNGS (+)	–
S114	Rhinovirus	–	tNGS (+)	–
S137	Influenza A virus	–	tNGS (+)	mNGS (+)

## 4 Discussion

NGS was initially proposed as a promising tool for pathogen identification in the early 2010s (Yongfeng et al., 2011; Miller et al., 2013), but has not gained popularity till recent years, particularly with the advent of mNGS, whose robustness in detecting respiratory pathogens has been demonstrated (Chen et al., 2020; Fang et al., 2020; Li et al., 2020; Shi et al., 2020; Jiang et al., 2021; Peng et al., 2021; Gaston et al., 2022; Miller et al., 2019). The emerging highly-multiplex-PCR-based tNGS has gained significant acceptance in China since the introduction of commercial products in 2021. In some prestigious hospitals, there have been arguments advocating for the implementation of tNGS and mNGS as routine testing procedures. While tNGS shows feasibility in diagnosing RTIs, its true value and efficacy still require solid evidence to be substantiated. In this study, we evaluated the pathogen detection performance of tNGS and mNGS based on the same clinical cohort of 180 enrolled BALF samples. Tests of tNGS and mNGS were conducted and reports were interpreted by two units. Pulmonologists evaluated the diagnosis value of pathogen reports case by case. Strict rules were set up during this stage, and all details were collected and taken into consideration to implement an objective, bias-free validation for both NGS approaches. Overall, the results demonstrated that tNGS had similar detection performance to mNGS although some variance was observed when identifying specific species. Both methods assist in etiology identification but tend to identify opportunistic pathogens with low clinical relevance.

In general, tNGS exhibited a high overall consistency with mNGS in detecting pathogens, as supported by the linearity (**Figure 2A**) and performance metrics (**Supplementary Table 4**). Small variance effect (standard median deviation = 0.288) and significance by Wilcoxon rank test implied that tNGS had a slightly higher chance of identifying organisms than mNGS, which was supported by higher detection rates per species (**Figure 2C**). This may be thanks to the pre-amplification procedure, which could overcome interference by high level of human genomic fragments and increase sensitivity to low abundant targets.

Unlike tNGS, additional workflow is required for mNGS to detect RNA targets, which doubles the cost and resource consumption. Due to the expense of mNGS and our initial focus not including RNA viruses, a total of 102 samples did not undergo mNGS-RNA test, among which tNGS identified RNA viruses in 13 samples. Regarding samples with both mNGS-RNA and tNGS reports, both methods detected 12 RNA species, indicating their similar abilities to identify RNA viruses, as most detection frequencies for each species were equal (**Supplementary Figure 2**).

The agreement between NGS outputs and clinical diagnosis is the key concern in this study. As a broad-spectrum molecular detection method, NGS tends to output a comprehensive list of microbes once the targets are detected. Such a list usually includes opportunistic pathogens which only cause diseases on hosts with immunocompromised conditions. The less likelihood for a pathogen causing observable symptoms, the less likelihood clinicians link it to casual factors. This may explain the low PPVs for all opportunistic pathogens (**Supplementary Table 5**). One extreme case is Human gammaherpesvirus 4, with PPVs of 2.17% and 3.23% for tNGS and mNGS, respectively. Meanwhile, four selected microbes, *Aspergillus fumigata*, *Chlamydia psittaci*, *Crptococcus neoformans*, and *Mycobacterium tuberculosis* had higher PPVs (66.7%~100%, **Table 3**), demonstrating high agreement on these organisms as “pathogen” in clinical prospect.

A classification system was established to assess the diagnosis value of NGS methods to clinical decision under different scenarios (**Figure 3**; **Table 1**). All reports were adjudicated and labeled as Agree, Extend/Support, Noncontributory, Unlikely, or Disagree to ensure an intuitive summary. An ideal method should output a higher percentage of “good” results, that is those reports with Agree or Support/Extend tags. Both tNGS and mNGS had a combined ratio of 65.6% (119/180) and 68.9% (124/180), indicating that NGS methods could assist in identifying potential pathogens. The cases labeled as ‘Support’ or ‘Extend’ had their etiology undetermined until the introduction of NGS, which enabled identification at the species level, reflecting NGS’s ability to improve etiology determination. Such cases accounted for 33.9% and 30.6% for tNGS and mNGS, respectively. These findings suggest that tNGS may enhance medical service similarly to mNGS, as illustrated in **Figure 3A**.

Cases categorized as Noncontributory, Unlikely, or Disagree for tNGS and mNGS, respectively revealed NGS’s limitations in some clinical cases. A preference of tNGS in Unlikely cases and mNGS in Disagree cases was observed (**Figure 3B**). We hypothesize that it might be due to the differences in sensitivity. The pathogen detection ability of mNGS was interfered by a high host genetic background (Miller et al., 2019) while the targeted amplification strategy makes tNGS avoid such interference and increase sensitivity. This could also apply to the Noncontributory cases, as a majority of the cases reported opportunistic pathogens, accounting for 13.9%/13.9% of all samples for tNGS/mNGS. This further supports our hypothesis that pathogenicity significantly influences the agreement between NGS results and clinical diagnosis. However, it is hard to conclude that NGS is Noncontributory. Firstly, pathogen reports at the early stage of infection could serve as an indicator of potential threat for immunocompromised patients with poor prognosis. Secondly, it is hard to exclude the role of opportunistic pathogens in exacerbating illness in cases of co-infection. In our opinion, both NGS methods are better suited as tools for profiling potential organism threats while it is the medical personnel’s responsibility to conclude the relevance of the threats. This holds true for any detection tool, not just NGS. Therefore, interpretation is critical when employing NGS in pathogen detection.

**Table 5** highlights several technical and practical considerations of mNGS and tNGS for pathogen identification. The tNGS panel focuses on specific pathogen species with high clinical relevance while mNGS detects all microbes without bias. Additionally, tNGS utilizes a panel design and pre-amplification strategy, resulting in high data usage and minimized data throughput requirement (100K for tNGS, 20M for mNGS). The cost of mNGS in this study ranged from 289.2 to 462.7 USD per assay (batch run), while tNGS was roughly a quarter of that cost. Moreover, regular mNGS requires additional nucleic acid extraction, library construction, and sequencing steps to detect RNA viruses, making the whole procedure of mNGS more complicated than tNGS. Overall, these advantages make tNGS a cost-efficient fit for clinical microbiology. The overall TAT of tNGS and mNGS were 12h and 17h, respectively, representing the internal time from sample preprocessing to report output. When combined with the delivery time, the total TAT may reach 1~2d, depending on the distance from sampling location to the third-party sequencing laboratory, which may delay the NGS report/service in meeting clinical needs. Delivery is one of the issues that NGS service providers need to improve through speeding up transportation by constructing an intensive network, or achieving sequencing laboratory localization. The laboratory--developed test (LDT) should be one of the trending solutions for NGS in the upcoming decade, allowing hospitals to develop LDT in place, which should significantly shorten the TAT in the future.

**Table 5:** Technical comparison of NGS in RTI diagnosis.

Features	mNGS	tNGS
Spectrum	Broad (target species > 20,000)	Broad (target species = 153)
Target Limit	Theoretically unlimited in sequencing level but pathogen calling limited to the reference database	Limited to the panel; may miss causal pathogens
Interference		
- high host genetic background	Major impact	Minor impact
- low-titer target species	Major impact	Minor impact
Simultaneous DNA + RNA Pathogen Identification	No, separate nucleic acid extraction, library and sequencing procedures are required for DNA and RNA pathogens, individually	Yes
Prior-knowledge Requirement	Yes, required downstream of sequencing (bioinformatics analysis step)	Yes, required prior to sequencing (enrichment step) bioinformatics analysis step
Prepossessing	Host depletion	Target amplification by multiplex PCR
Processing and Library Construction	Relatively more complicated processing procedures, more reagent cost (human depletion; additional extraction for RNA library)	Simple procedure
Average Output Size for Qualified Report	20 million reads	100 kilo reads
Effective Data %	Low, require host depletion	High
Interpretation	Required to determine potential pathogens from a comprehensive list, markedly more pathogens require interpretation	Fewer pathogens reported, but still requires interpretation
Turn Around Time	~17 h	~12 h
Cost per Assay/Patient	mNGS(DNA): ~600 USD	~120 USD
	mNGS(DNA+RNA): ~1000 USD	(pending on panel)

It’s hard to conclude that tNGS is a substitute for mNGS in pathogen identification. The specificity and target spectrum of a multiplex-PCR-based tNGS largely depend on the primer set design. In this study, a total of 28 species were missed by the tNGS panel, as summarized in **Supplementary Table 3**. However, only 3 of them were clinically significant. The inability of tNGS to identify unknown pathogens could be problematic when an acute infection is caused by new/unknown pathogens, e.g. SARS-COV-2 pandemic in 2019. Although the spectrum limit of tNGS could be resolved by increasing target species, the difficulty of primer set design significantly increases, and pre-amplification may enhance contamination signals, posing another challenge to tNGS application. Thus, the decision to use either mNGS or tNGS should be guided by several factors, including the clinical scenario, technical distinctions, emergence, and cost. For cases requiring rapid identification of an etiology, tNGS is recommended due to its shorter turnaround time (TAT). In contrast, for severe infections where a comprehensive report on pathogens and drug resistance is crucial, mNGS may be prioritized over tNGS, given its broader target spectrum. In some situations, employing both mNGS and tNGS concurrently might be beneficial. Moreover, for cases where there is a suspicion of new or unknown pathogens, or where routine clinical laboratory tests have returned negatives or have an inadequate spectrum, mNGS is recommended. While this study only included bronchoalveolar lavage fluid (BALF) samples, it is important to note that other sample types, including more complex samples like formalin-fixed, paraffin-embedded (FFPE) tissues or blood samples with low host genetic material, could also be effectively analyzed. This approach ensures that the choice between mNGS and tNGS is aligned with the specific needs of the clinical context, leveraging each method’s strengths to optimize pathogen identification and patient outcomes.

A recent study compared the performance of tNGS and mNGS in RTI diagnosis on the same cohort (Sun et al., 2024), which employed a similar design as ours. Both Sun’s study and this study remonstrated that the overall performance of tNGS and mNGS in pathogen detection was relatively similar while significant differences were noted in the detection of specific pathogens. For Human gammaherpesvirus 4, Human betaherpesvirus 7, and Human betaherpesvirus 5, which were focal points of Sun’s study, our findings indicate significant differences between tNGS and mNGS (P<0.05) and tNGS presented higher detection frequencies for all these viruses, aligning with the Sun’s study. Conversely, in the case of Human betaherpesvirus 6, our analysis identified only three positives by tNGS whereas mNGS yielded non, thus it could not be directly compared to Sun’s result (12 verse 4). Nonetheless, it’s also difficult to conduct direct parallel comparison regarding other pathogens. First, Sun’s study employed a relatively small sample size of 80, which is hard to confirm such sample size is robust enough for reliable conclusions, especially for the pathogens with low detection rates (25 species had only 1 positive in Sun’s study), let alone Sun’s study didn’t involve mNGS-RNA procedures. In contrast, our study was informed by prior exploratory research and adopted a sample size exceeding 190 to ensure robustness (demonstrated in Methods and materials 2.8). Secondly, the sensitivity and specificity of pathogen detection are heavily contingent upon the primer sets utilized. Without transparency of primer settings in these commercial tNGS panels in these two studies, it is difficult to critically evaluate these differences. Thirdly, our study opted for simultaneous application of both mNGS and tNGS, whereas the other study subjected residual mNGS samples to freeze-thaw cycles before conducting tNGS, which could have inadvertently compromised the sensitivity of tNGS for certain samples. Also, Our study design did not include an orthogonal validation, like the retrospective PCR in Sun’s study, since we have already employed a composite reference standard, which takes results from single to multiple prior microbial testings for robust etiology.

In conclusion, this study demonstrates the robustness of a highly-multiplex PCR based tNGS method in respiratory pathogen detection, as an economical alternative to mNGS. Although mNGS offers theoretically unlimited sequencing depth, it requires a higher cost per assay than tNGS. Conversely, tNGS is more cost-effective and less susceptible to inference impact, but restricted to specific panel targets. The sequencing methods, therefore, should be selected based on specific goals, available resources, and trade-offs between sensitivity, specificity, and cost. Our recommendation is to prioritize tNGS as a primary option for RTI diagnosis due to its lower cost, while keeping mNGS available as an alternative if tNGS proves inadequate in clinical practice. Although tNGS and mNGS are complementary in pathogen detection, reliable results must be obtained in combination with sophisticated interpretation and adjudication. Validating the clinical relevance of NGS reports requires taking into account the pathogenicity of the reported organisms and low-abundance candidates. Overall, these findings offer valuable insights for decision-making in microbial identification and can assist researchers in optimizing the use of sequencing technologies for medical purposes.

## Data Availability

All sequencing data generated for this study, including tNGS and mNGS, have been deposited in the Genome Sequence Archive (Chen et al., 2021) database of National Genomics Data Center under the accession number PRJCA018285.
