# Supplementary material for: Diagnosis value of targeted and metagenomic sequencing in respiratory tract infection
Source: Front Cell Infect Microbiol. 2024 Dec 12;14:1498512. doi: 10.3389/fcimb.2024.1498512 (PMC11669359; doi:10.3389/fcimb.2024.1498512)
Supplement: Supplementary file 2 [file DataSheet2.docx]

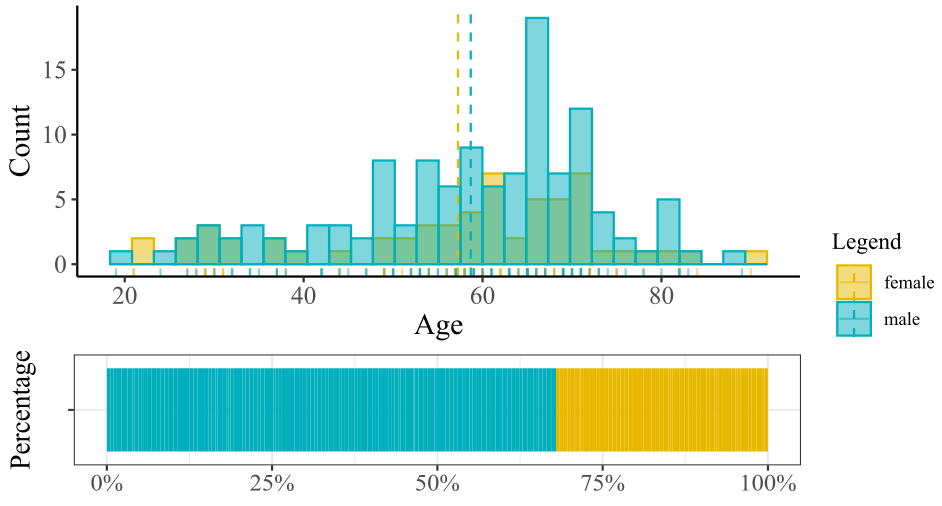
 **Supplementary Figure 1 Age and gender distribution of the study population**


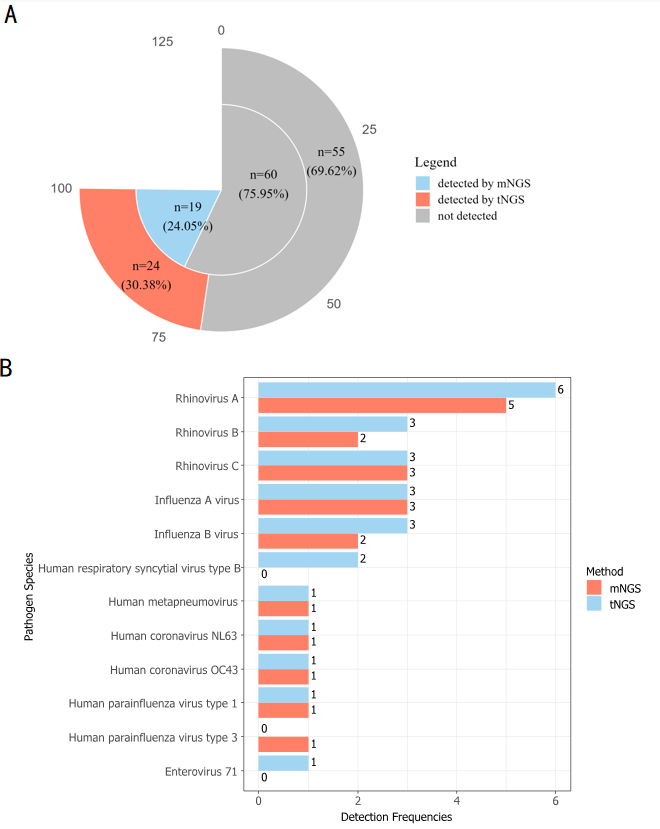


**Supplementary Figure 2 Method-wise comparison of the detection frequency of RNA viruses**
